# Cognitive Flexibility Training in Virtual Reality for Patients with Obsessive-Compulsive Disorder

**DOI:** 10.1192/j.eurpsy.2026.11008

**Published:** 2026-07-31

**Authors:** K. Zuzánková, M. Janíková, P. Stopková, I. Fajnerová

**Affiliations:** 1 Center for Virtual Reality Research in Mental Health and Neuroscience, National Institute of Mental Health, Klecany; 2 3rd Faculty of Medicine, Charles University, Prague, Czech Republic

## Abstract

**Introduction:**

Obsessive-compulsive disorder (OCD) is a persistent psychiatric condition displaying a variety of symptoms, defined as obsessions - intrusive thoughts eliciting anxiety, and compulsions - repetitive actions providing temporary relief from the anxiety provoked by the obsessions (Fontenelle et al., 2006). Recent neuropsychological evaluations indicate that OCD patients may have compromised cognitive flexibility (Gruner and Pittenger, 2017), and it is plausible to use training methods to target this impairment (Zuzankova & Fajnerova, 2023).

**Objectives:**

This feasibility study evaluates the integration of Virtual Reality-based Cognitive Training (VRCT) into inpatient cognitive-behavioral therapy (CBT) for obsessive-compulsive disorder (OCD). The primary aim is to assess participant engagement, adherence, and preliminary cognitive outcomes to determine the acceptability of VRCT in clinical settings.

**Methods:**

Cognitive flexibility (CF) and symptom severity will be measured through cognitive tasks (Trail Making Test, Stroop Task, Verbal Fluency, Go/NoGo Task, and the Berg’s Card Sorting Test (BCST) and self-report scales (CFI, Y-BOCS). Feasibility metrics include adherence rates, task completion, and subjective experience of presence and cybersickness (IPQ, SUS, SSQ). The study involves 25 participants with OCD (F42 in ICD-10; 6B20 in ICD-11) undergoing a 6.5-week CBT program with VRCT.

**Results:**

Analyses will include descriptive statistics, correlation analyses, and thematic analysis of participant feedback.

**Conclusions:**

This study provides insights into the acceptability and practicality of VRCT for OCD treatment, laying the groundwork for future research on its clinical efficacy.

**Disclosure of Interest:**

None Declared

